# Amplitude of Low-Frequency Fluctuations and Resting-State Functional Connectivity in Trait Positive Empathy: A Resting-State fMRI Study

**DOI:** 10.3389/fpsyt.2021.604106

**Published:** 2021-02-18

**Authors:** Tong Yue, Jia Zhao, Anguo Fu

**Affiliations:** ^1^Faculty of Psychology, Southwest University, Chongqing, China; ^2^School of Management, Hainan University; Hainan Institute of Corporate Governance, Haikou, China

**Keywords:** trait positive empathy, amplitude of low-frequency fluctuations, resting-state fMRI, functional connectivity, self

## Abstract

Positive empathy is the ability to share and understand the positive emotions of others. In recent years, although positive empathy has received more and more attention, trait positive empathy (TPE)-related spontaneous brain activity during the resting state has not been extensively explored. We used the amplitude of low-frequency fluctuations (ALFFs) and resting-state functional connectivity (RSFC) of the resting-state functional magnetic resonance imaging signal to explore TPE-associated brain regions. We found that higher TPE was associated with higher ALFFs in the right insula and lower ALFFs in the right subgenual cingulate (SGC), right dorsomedial prefrontal cortex (dmPFC), and right precuneus. RSFC analyses showed that higher functional connectivity between the right insula and left parahippocampal gyrus, left inferior parietal lobule and left middle temporal gyrus were related to higher TPE. Moreover, the connection between the right dmPFC and the left medial orbitofrontal cortex, left middle occipital gyrus and left posterior cingulate cortex were positively related to TPE. Meanwhile, the strength of functional connectivity between the right SGC and left supplementary motor area was positively associated with TPE. These findings may indicate that TPE is linked to emotional (especially the experience of more positive emotions and better negative emotion regulation) and self-referential processing.

## Introduction

Empathy is the ability to generate an isomorphic affective state by inferring the affective states of others while retaining self-other awareness (1). As an important psychological ability, it is a critical component of the social interactions of our complex social world. Different individuals have different levels of empathy, and this personality difference in trait empathy has always an important area of research.

In recent years, researchers have also begun to explore the neurological bases of individual differences in trait empathy. Using resting-state functional magnetic resonance imaging (fMRI), researchers can examine the spontaneous activity of brain function without external information input. They can then explore how the differences in trait empathy are reflected in intrinsic neural activity. For example, Cox et al. (2) measured trait empathy in healthy subjects using the interpersonal reactivity index (3) to study how empathic ability is reflected in the brain. Their results indicate that affective empathy ability is associated with stronger functional connectivity among social-emotional regions, such as the ventral anterior insula, orbitofrontal cortex, amygdala, and perigenual anterior cingulate, which have critical putative functions in experiencing and understanding others' emotions. Using empathizing quotients (EQ) (4), Takeuchi et al. (5) investigated empathizing-related resting-state functional connectivity (RSFC) in 248 healthy young adults. They found that higher empathy was associated with increased RSFC between the medial prefrontal cortex and precuneus/middle cingulate cortex, all of which are key nodes of the default mode network (DMN). The author argues that this illustrates the important role of the DMN in empathy, as individuals with higher levels of trait empathy may be better at using the medial prefrontal cortex and precuneus/middle cingulate cortex to speculate others' emotional state.

In general, the aforementioned studies have deepened our understanding of trait empathy based on neural activity levels. However, previous studies have focused primarily on empathy as a trait relevant to the negative spectrum of emotions (e.g., empathy for distressed and sad individuals or negative empathy) (6–8). Although the interpersonal reactivity index and empathizing quotient are widely used instruments in trait empathy research, most of their related items measure the individual's feelings of negative emotions. Therefore, there are great limitations on the use of the above questionnaires to explore the neural mechanism of trait empathy because they in fact explore individual differences in trait negative empathy (TNE), as reflected in brain activity.

In fact, empathizing with others' positive emotions is also an important part of our lives. The ability to share and understand the positive emotions of others is defined as positive empathy (7, 9), which is a new field in empathy research, although it has received increased attention in recent years. With the help of measuring tools created to assess trait positive empathy (TPE), such as the Positive Empathy Scale (PES) (7) and the Dispositional Positive Empathy Scale (9), researchers have found that TPE and TNE might be two similar but independent psychological structures. TPE is highly correlated with empathic concern (7, 9, 10), which is an important component of TNE as assessed using the interpersonal reactivity index. This indicates that individuals with higher levels of TPE are also more sensitive to others' emotional information, such as TNE. In contrast, trait positive empathy is associated with lower levels of dispositional negative emotionality and higher levels of dispositional positive emotionality, but this trend is reversed in TNE (7, 10). Based on these observations, Yue et al. (11) recently tentatively explored the neural mechanism underlying TPE using a voxel-based morphometry analysis. The results indicated that the ability to exhibit TPE is positively correlated with the volume of gray matter in the right insula, left anterior cingulate cortex, dorsolateral prefrontal cortex, and medial prefrontal cortex. We can conclude that emotion processing and regulation may play vital roles in the differences in TPE. In brief, the above studies explored the psychological characteristics of TPE from different perspectives and deepens our understanding of this personality trait.

To our knowledge, there no study has investigated TPE-related spontaneous brain activity during the resting state until now. According to the basic principle of the amplitude of low frequency fluctuations (ALFF) analysis (12), which assumes that the brain BOLD signal has physiological significance in the low-frequency range, the average value of amplitudes at all frequency points within a frequency band (0.01–0.08 Hz) is used to characterize the strength of a voxel's spontaneous activity. ALFF reflects the level of spontaneous activity of each voxel from the perspective of energy, and has been shown to correlate with many personality traits (13–15). RSFC may be used to measure synchronization between different regional spontaneous neuronal signals and reflect the characteristics of the co-activated cortical networks (16, 17). In previous studies using RSFC, researchers have explored many personality trait-related brain regions or networks to study autistic traits (18), risk propensity (19), and Big Five personality (20, 21).

In this study, ALFFs and the RSFC of the resting-state fMRI signals were used to explore TPE-related brain regions and networks during spontaneous brain activity in the resting state. Previous studies have shown that subjects with higher levels of TPE may have better emotion regulation abilities, especially in positive emotional information processing and negative emotional information regulation (7, 10, 11). Thus, we hypothesized that TPE is linked to brain networks related to emotional processing and regulation. In addition, positive emotional sharing involves understanding and speculation regarding the mental states of others. Therefore, the DMN may also play an important role in this process. We hypothesized that the above brain regions and networks may work together to subserve TPE.

## Methods

### Participants

We recruited 87 undergraduate and graduate students via advertisements posted in the Bulletin Board System of Southwest University, and six participants were excluded from the sample owing to excessive head movement (see the Data Preprocessing section). The participants were all healthy and right-handed. Using a self-reporting inventory, we confirmed that none of the participants in this study had a history of neurological illness. After the experiment, the participants were required to complete the positive empathy scale (see below for details) immediately. They were then paid and thanked for their participation. The analyzed data consisted of information from 39 women and 42 men whose average age was 21.38 ± 2.00 years. The ages of the participants ranged from 18 to 26 years.

This study was approved by the Ethics Committee of Southwest University and conformed to the tenets of the Declaration of Helsinki. Before experimentation, we informed the participants regarding their privacy rights and that they could quit the experiments at any time. The participants were also asked to provide written consent and were paid $8 for their participation after the experiments.

### Assessment of TPE

We used the Chinese version of PES (22) to assess the participants' TPE, which includes seven items, such as “When I see someone else smile, I can't help but smile, too.” A 5-point Likert scale was used for this questionnaire, ranging from “1 = does not describe me at all” to “5 = describes me very well”, higher scores represent higher tendency to share, celebrate, and enjoy others' happiness. The Chinese version of PES has been reported to have strong internal reliability and temporal stability, with a Cronbach's alpha of 0.84 and a test-retest reliability coefficient of 0.78. In this study, the Cronbach's alpha was 0.837.

### Image Acquisition

A 3.0 Tesla Siemens Trio scanner (Siemens Medical; Erlangen, Germany) in Southwest University was used to scan the participants in this study. We fixed the participant's head position using foam to reduce head movement. Functional MR images were acquired using a single-shot, gradient-recalled echo planar imaging sequence (repetition time = 2,000 ms, echo time = 30 ms, flip angle = 90, 32 axial slices, field of view = 200 × 200 mm, acquisition matrix = 64 × 64, slice thickness = 3 mm, voxel size = 3 × 3 × 3 mm), slice distance = 0.6 mm. The participants were asked to rest in the scanner with their eyes closed simply, and not to sleep or think of anything. We acquired 8 min of resting data from each participant. We then used a three-dimensional magnetization prepared rapid gradient echo sequence (176 slices, repetition time = 1,900 ms, echo time = 2.52 ms, flip angle = 9°, resolution = 256 × 256, and voxel size = 1 × 1 × 1 mm) on each participant for spatial normalization and localization to acquire high-resolution T1-weighted anatomical images in the sagittal orientation.

### Data Pre-processing

In this study, we used Data Processing Assistant for Resting-State fMRI (DPABI, 2.3, Advanced edition) to pre-process the data. The following steps were carried out: (1) removing the first 10 volumes of the 230 volumes to ensure steady-state magnetization for final analysis; (2) slice timing; (3) correcting for head movements, which required the images to be realigned with a six-parameter (rigid body) linear transformation and the removal of 6 participants who had head motion >2-mm maximal displacement; meanwhile, the frame-wise displacement (FD) with Jenkinson algorithm is calculated for the all of the data, where the residual data (81 participants) satisfied the FD criteria (FD < 0.2) with group mean and standard deviation as 0.05959 ± 0.02306; (4) segmentation of T1-weighted images into gray matter, white matter, and cerebrospinal fluid; (5) regressing out of 27 nuisance covariates, including signals from white matter and, cerebrospinal fluid, global signals, and Friston 24 motion parameters; (6) spatial normalization to the MNI template; (7) resampling of images into a spatial resolution of 3 × 3 × 3 mm; (8) spatial smoothening with a Gaussian kernel of 6 mm full-width at half-maximum.

### Data Analysis

#### ALFF Calculations

The ALFF analysis was carried out using DPABI. First, Fourier transforms of each voxel of the whole brain was carried out to obtain the power spectrum in the frequency domain. The peak area of the power spectrum can be regarded as the energy of the signal. The power spectrum's square root was calculated and averaged across 0.01–0.08 Hz for each voxel. We then averaged the square roots of these values and obtained the ALFF (12). It should be stated here that ALFF is chosen instead of fALFF because it has higher test-retest reliability in gray matter regions than fALFF (23). In order to standardize our data, the ALFF value of each voxel was subtracted from the average ALFF value of the whole brain and divided by the standard deviation to finally obtain the normalized ALFF value of each voxel.

#### ALFF-Behavior Correlation Analysis

We carried out multiple regression analysis using the mask of the whole brain. We used PES score as the independent variable and ALFF value as the dependent variable. Simultaneously, sex, age, and years of education were included as covariates to eliminate the effects of these factors on outcomes. Multiple regression analysis results were corrected using AlphaSim program in the AFNI software (http://afni.nimh.nih.gov/pub/dist/doc/program_help/AlphaSim.html), which is based on a Monte Carlo simulation for correction of multiple comparisons. The threshold was set to a corrected cluster two-tailed *P* < 0.05 (single voxel *P* < 0.001, cluster size = 90 voxels [1,512 mm^3^], and 1,000 iterations). We enhanced the individual voxel threshold to *p* < 0.001 in order to increase the spatial specificity and better prevent the false positives for the corrected results (24–27).

#### Functional Connectivity Analysis

In order to further test whether the significant brain regions found in the ALFF- correlation analysis and other brain regions form a neural network to predict the level of TPE, we performed functional connectivity analysis. First, the above significant brain regions were used as regions of interest. The functional connectivity between these regions of interest and other voxels was calculated for the whole brain. We then calculated the strengths of the connections between these functions and TPE to further test whether there is a specific connection in these networks that can significantly predict TPE levels. The software used for functional connection analysis was REST 1.8. Before the formal analysis, the white matter signals, cerebrospinal fluid signals, whole brain mean signals, and head movement parameters (28) were regressed out. The specific calculation process was as follows: (1) calculate the mean time series for each participant's regions of interest; (2) obtain correlations between these time series and all-time series of other voxels in the gray matter mask; (3) using Fisher's r-Z transformation, convert the correlation maps produced in the above analysis to Z-maps; and (4) use a single-sample *t*-test to determine brain regions that were significantly associated with the seeds. Multiple comparisons correction was performed using Alphasim (corrected two-tailed *P* < 0.05, single voxel *P* < 0.001). The significant regions were then defined as masks. We assessed the RSFC-behavior correlations in these masks to examine the functional connectivities that were correlated with PES. All of the results were corrected in the corresponding masks using AlphaSim.

## Results

### PES-Related Brain Regions

**Figure 1:**
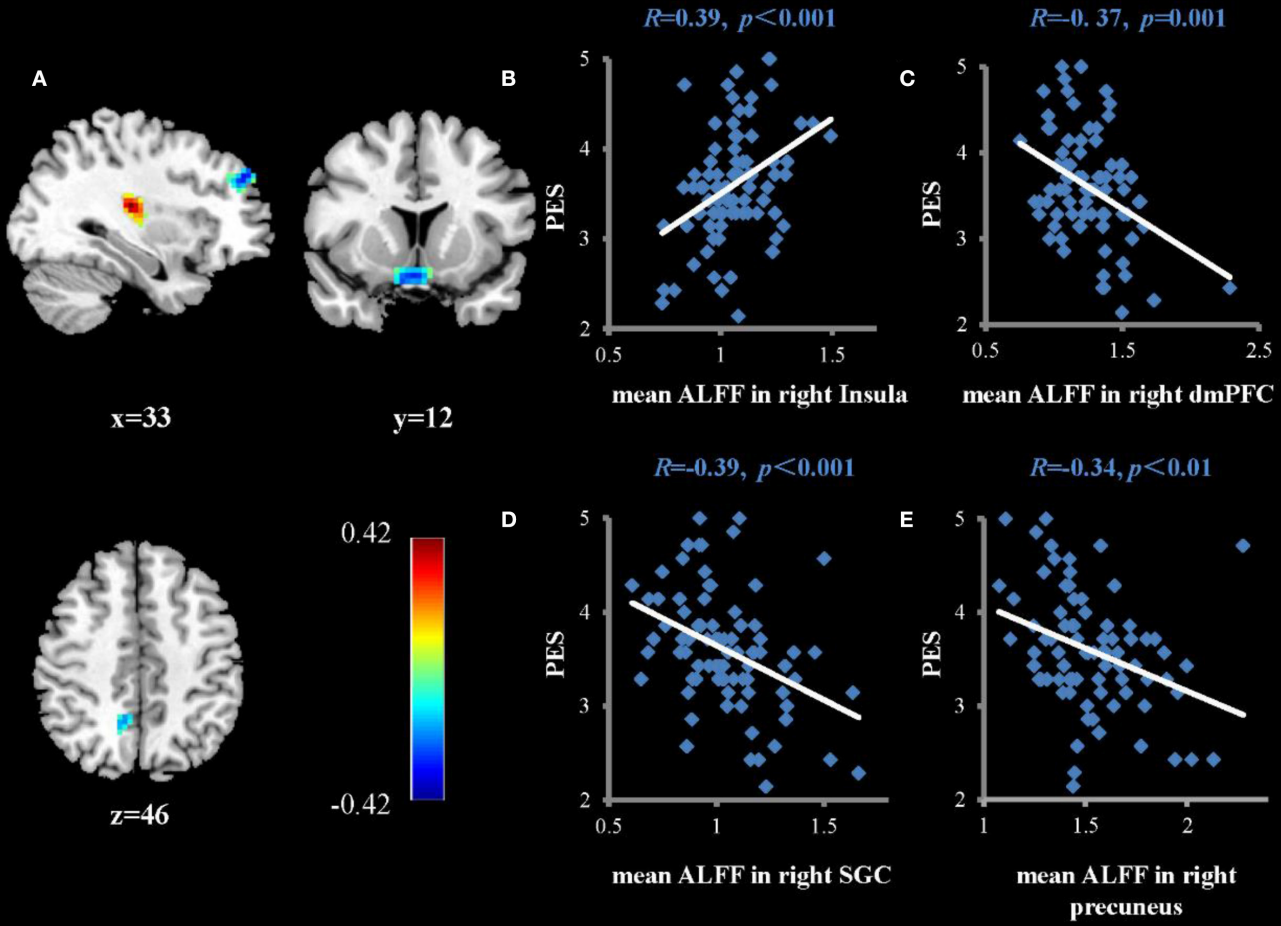
Brain regions that show significant correlations between ALFFs and the average score of PES **(A)** (multiple regressions, *P* < 0.05, AlphaSim-corrected). Color bars represent R-values. L = left, R = right. **(B–E)** show scatterplots of the correlations between PES score and mean ALFFs in the right insula, dorsomedial prefrontal cortex (dmPFC), subgenual cingulate (SGC), and right precuneus, respectively.

Regression analysis was used to investigate the association between TPE and the ALFFs of the resting state of the brain. Factors that may have influenced TPE (age, sex, and years of education) were regressed as nuisance covariates. As shown in Table 1 and Figure 1, PES scores were positively correlated with ALFFs in the right insula (*r*_peak_ = 0.42, *r*_cluster_ = 0.39, *p* < 0.001) and negatively correlated with ALFFs in the right subgenual cingulate (*r*_peak_ = −0.36, *r*_cluster_ = −0.36, *p* < 0.001), right dorsomedial prefrontal cortex (*r*_peak_ = −0.40, *r*_cluster_ = −0.30, *p* < 0.001), and right precuneus (*r*_peak_ = −0.34, *r*_cluster_ = −0.34, *p* < 0.01).

**Table 1:** Regions in which ALFFs were significantly related to PES.

**Brain regions**	**Side**	**BAs**	**MNI**			**Voxel size**	**Peak R**
			**x**	**y**	**z**		
Insula	R	13	33	−24	15	91	0.39
Dorsomedial prefrontal cortex	R	9	36	45	33	125	−0.37
Subgenual cingulate	R	25	3	12	−18	228	−0.36
Precuneus	R	7	9	−51	48	134	−0.33

*The columns provide anatomical descriptions of the regions of interest: hemisphere side (B: bilateral; R: right; and L: left), Brodmann areas (BAs), the peak Montreal Neurological Institute coordinate, the volume in voxels, and the peak R-value*.

### Functional Network Associated With TPE

**Figure 2:**
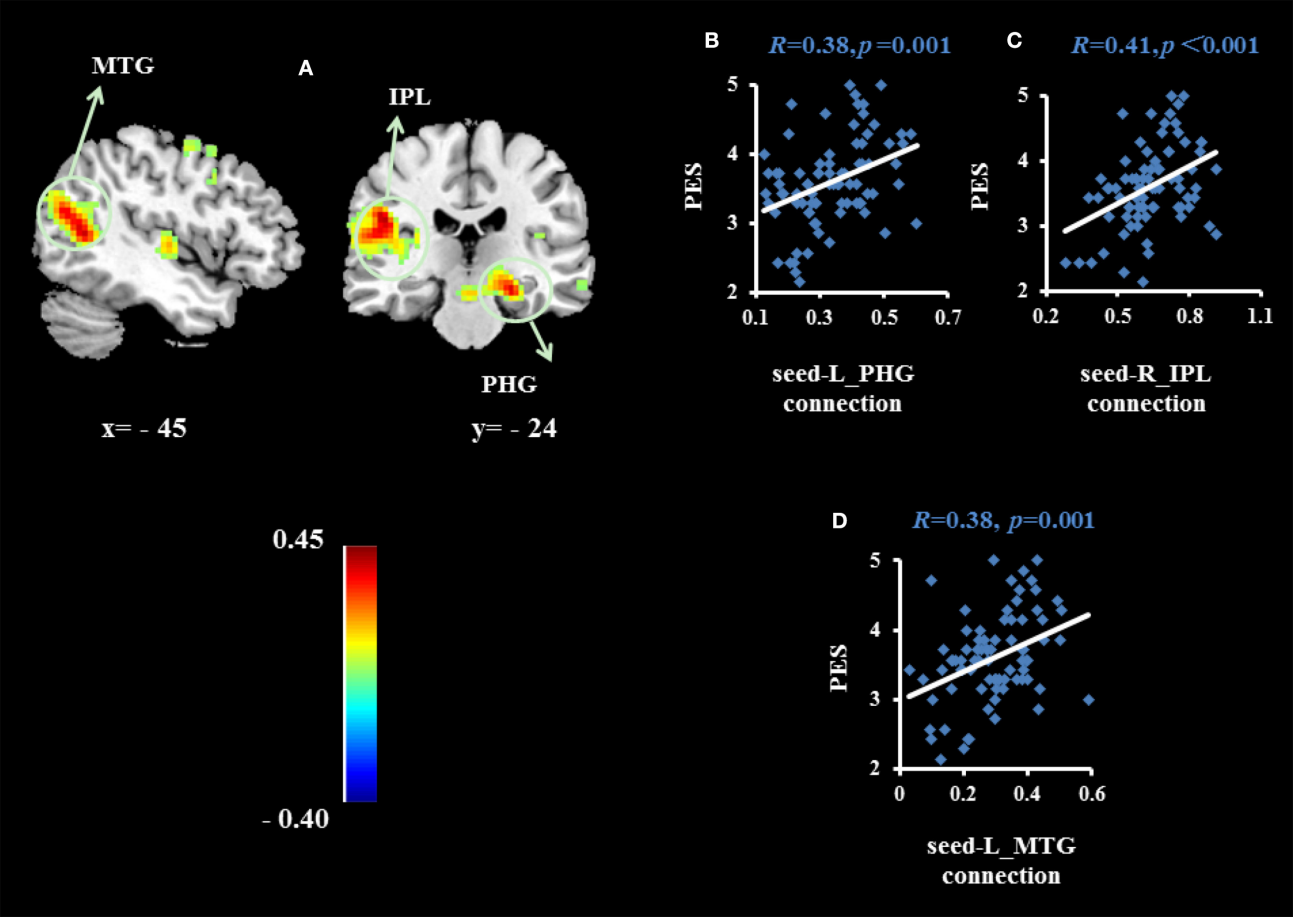
Brain regions whose functional connectivity strengths with the right insula (seed) were significantly associated with the average score of PES **(A)**. The threshold of corrected cluster was set at *P* < 0.05 [single voxel *P* < 0.001, cluster size > 226 voxels, and 1000 iterations]. Color bars represent R-values. L = left, R = right. **(B–D)** indicate significant correlations between PES and functional connectivity strengths between the left parahippocampal gyrus (PHG), left inferior parietal lobule (IPL) and left middle temporal gyrus (MTG).

**Figure 3:**
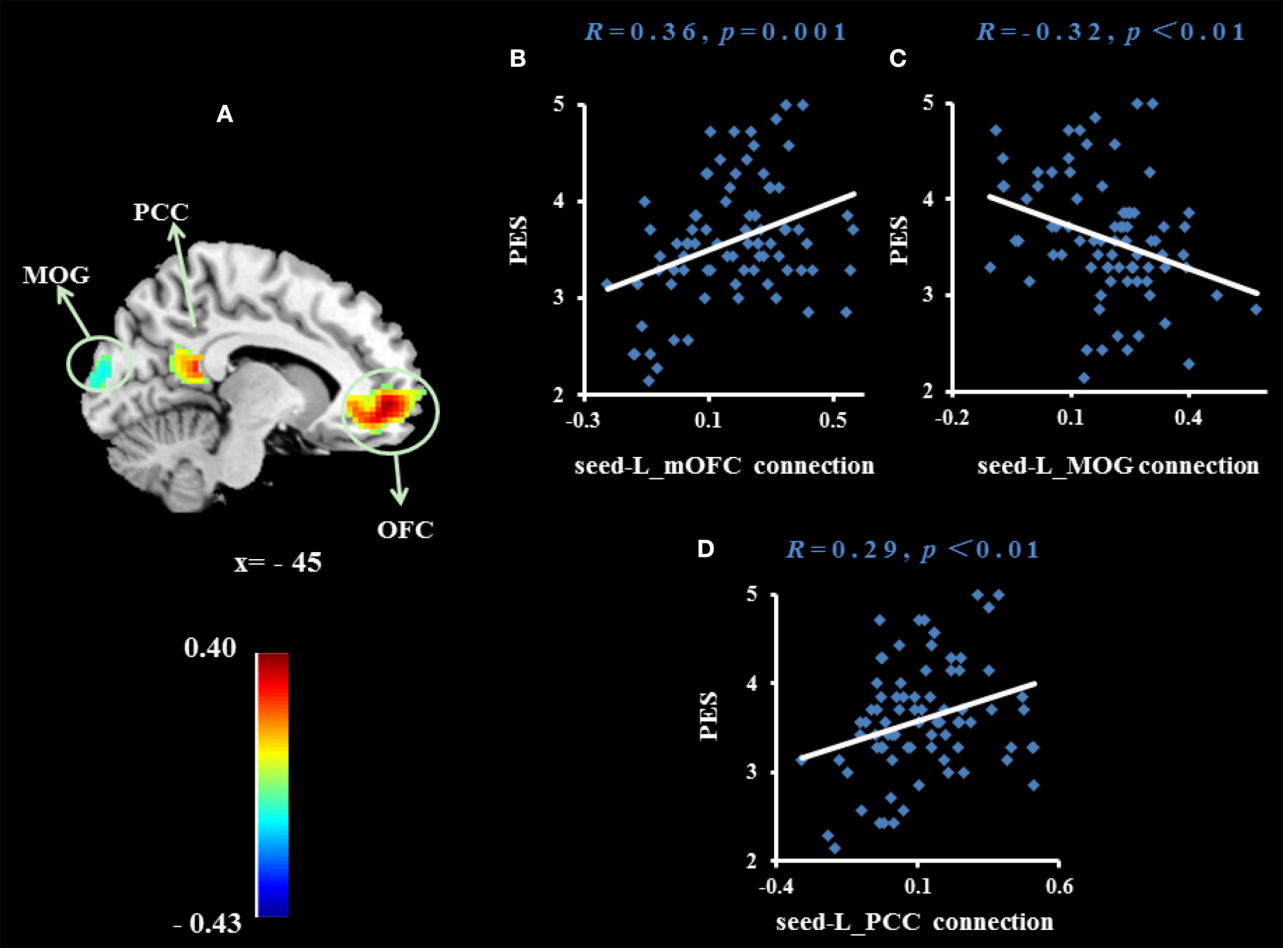
Brain region whose functional connectivity strength with the right dorsomedial prefrontal cortex (seed) was significantly associated with the average score of PES **(A)**. The threshold of corrected cluster was set at *P* < 0.05 [single voxel *P* < 0.001, cluster size > 137 voxels, and 1000 iterations]. Color bars represent R-values. L = left, R = right. **(B–D)** Indicate significant correlations between PES and functional connectivity strengths between the left medial orbitofrontal cortex (mOFC), left middle occipital gyrus (MOG) and left posterior cingulate cortex (PCC).

**Figure 4:**
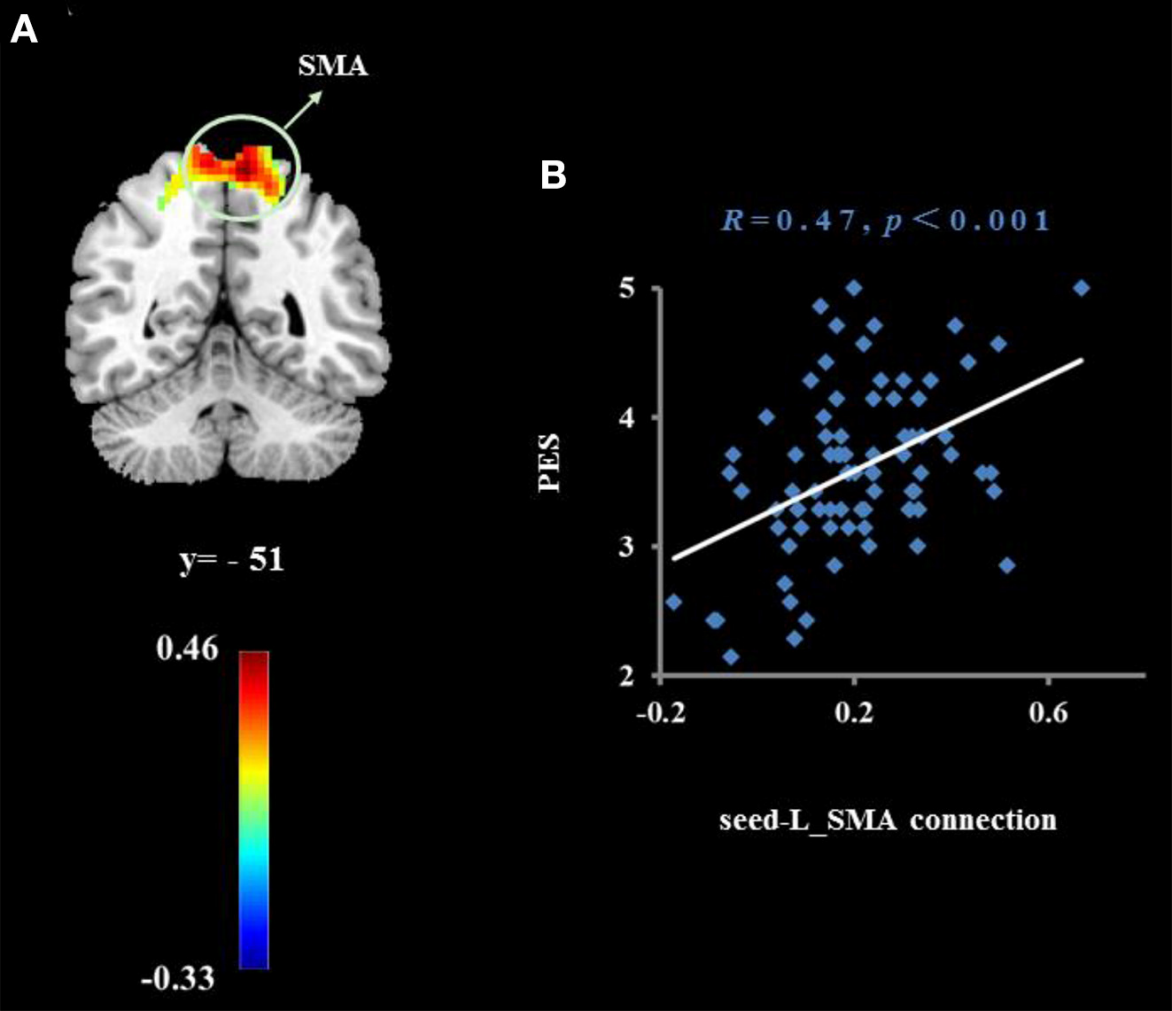
Cluster whose functional connectivity strength with the right subgenual cingulate (seed) was significantly associated with the average score of PES **(A)**. The threshold of corrected cluster was set at *P* < 0.05 [single voxel *P* < 0.001, cluster size > 276 voxels, and 1000 iterations]. Color bars represent *R*-values. L = left, R = right. **(B)** indicates a significant correlation between the average score of PES and functional connectivity strength with the left supplementary motor area (SMA).

To examine whether the regions that were observed in the ALFF-behavior correlation analysis work in concert with other regions as a network that correlate with TPE, we conducted a functional connectivity analysis. As shown in Table 2 and Figures 2–4, we found that the strengths of functional connectivity between the right insula and the following regions were significantly associated with PES: left parahippocampal gyrus (*r*_peak_ = 0.40, *r*_cluster_ = 0.38, *p* = 0.001), left inferior parietal lobule (*r*_peak_ = 0.45, *r*_cluster_ = 0.41, *p* < 0.001) and left middle temporal gyrus(*r*_peak_ = 0.41, *r*_cluster_ = 0.38, *p* = 0.001). With the right dorsomedial prefrontal cortex as the seed region, the strength of functional connectivity between this seed and the left medial orbitofrontal cortex (*r*_peak_ = 0.41, *r*_cluster_ = 0.36, *p* = 0.001), left middle occipital gyrus (*r*_peak_ = −0.38, *r*_cluster_ = −0.32, *p* < 0.01) and posterior cingulate cortex (*r*_peak_ = 0.34, *r*_cluster_ = 0.29, *p* < 0.01) were significantly related to PES. With the right subgenual cingulate as the seed region, the strength of functional connectivity between it and the left supplementary motor area (*r*_peak_ = 0.46, *r*_cluster_ = 0.47, *p* < 0.001) was significantly related to PES. No significant results were identified using this analysis method when considering the right precuneus as the seed.

**Table 2:** Brain regions in functional connectivity strengths with seeds were significantly related to PES.

**Brain regions**	**MNI**			**Peak**	**Voxel**
	**x**	**y**	**z**	**R**	**Size**
**R insula as the seed**					
L parahippocampal gyrus	−24	−24	−15	0.4	556
L inferior parietal lobule	51	−21	15	0.45	457
L middle temporal gyrus	−45	−60	9	0.41	482
L medial orbitofrontal cortex	−6	51	−6	0.41	528
L middle occipital gyrus	−27	−99	3	−0.38	195
L posterior cingulate cortex	−6	−45	15	0.34	209
**R subgenual cingulate as the seed**					
L supplementary motor area	−9	−52	69	0.46	664
**R precuneus as the seed**					
None significant					

*The columns provide anatomical descriptions of the regions of interest: the peak Montreal Neurological Institute coordinate, the volume in voxels and peak r-value*.

## Discussion

Here we performed ALFF-PES correlation analysis and seed-based RSFC analysis to investigate the neural correlates of TPE. The results indicate that ALFF and RSFC reflect individual differences in TPE during the resting state. Specifically, we found that higher PES was associated with higher ALFFs in the right insula and lower ALFFs in the right subgenual cingulate, right dorsomedial prefrontal cortex, and right precuneus. RSFC analysis further revealed that higher functional connectivity between the right insula and left parahippocampal gyrus, left inferior parietal lobule and left middle temporal gyrus are associated with higher PES. Moreover, the strengths of functional connectivity between the right dorsomedial prefrontal cortex and the left medial orbitofrontal cortex, left middle occipital gyrus and left posterior cingulate cortex were positively related to PES. The connection between the right subgenual cingulate and left SMA was positively associated with PES.

The ALFFs in the right insula were positively associated with PES scores in our study, indicating that individuals with higher levels of TPE have higher spontaneous neural activity in this region. The results of two recent meta-analyses of neuroimaging research on empathy (mainly focused on empathy for pain) found that the anterior insula plays an important role in vicariously sharing many emotions and sensations (29, 30). According to Craig and Craig (31)'s model, the physiological state of the body may be mapped to the posterior insula and then represented in the anterior insula. The above process may be used by individuals to experience subjective affection. This would enable individuals to integrate their interoceptive states into global feelings and modulate their adaptive behavior according to their empathic states (32). In summary, this phenomenon may reflect a common feature underlying empathy ability, wherein individuals who have high levels of positive empathy are also sensitive to emotional information from others.

The strengths of functional connectivity between the right insula and the left parahippocampal gyrus, left inferior parietal lobule and left middle temporal gyrus were significantly associated with PES scores when the right insula was used as the seed region. The parahippocampal gyrus has been associated with self-related processing, social processing, and emotional simulations of hypothetical social scenarios in previous studies (33). The inferior parietal lobule and middle temporal gyrus have been shown to be involved in emotional empathy (34–36). The inferior parietal lobule is one part of the human mirror neuron system, a network of brain regions which are involved in the perception of actions from others (34, 37, 38), which were mainly involvement in the empathic processes such as emotional empathy (39) and emotional imitation (34). Thus, the observed activity of inferior parietal lobule may subserve emotion regulation progress. The middle temporal gyrus has also been shown to be involved in emotional empathy in previous studies (34–36). Its main function is to perceive social situations (40) and to infer the mental states of others (41). Our results have shown that the above brain areas and their connections with the right insula are positively related to TPE. It is likely that individuals with higher levels of trait positive empathy are more adept at extrapolating others' mental states and can effectively understand and share the emotions of others by recalling their own emotional experiences.

We also found that ALFFs in the right dorsomedial prefrontal cortex and right precuneus were negatively correlated with PES scores. The precuneus is an important structure of the DMN, and is generally considered to be involved in self-related episodic memory and self-reflection (42). Previous resting fMRI studies on depression have found that functional connections of the precuneus in patients with depression are higher than they are in healthy subjects. Thus, individuals with low happiness have relatively high ALFF values in this brain area. This may indicate that these individuals excessively think about themselves. The dorsomedial prefrontal cortex may be involved in the cognitive control network, the DMN, and the affective network (43). It is often involved in self-referential and emotional processing (33) and is a core region of the DMN. In clinical research, patients with depression often display hyperconnectivity of the dorsomedial prefrontal cortex when compared to healthy subjects (43, 44). For example, patients with the first untreated episode of depression have abnormal increases in regional homogeneity in the resting state in the dorsomedial prefrontal cortex. This may be associated with excessive self-care or self-concern (45). Taken together, the higher ALFFs in the right dorsomedial prefrontal cortex and right precuneus in subjects with lower positive empathy may indicate that these individuals excessively think about themselves, have a self-centered way of thinking, or have difficulties sharing the positive emotions of others. This may further illustrate an important psychological characteristic of individuals who are adept at sharing the positive emotions of others: lower self-centered tendencies.

When we used the right dorsomedial prefrontal cortex as the seed region, we found that the strength of functional connectivity between this seed and the left medial orbitofrontal cortex, left middle occipital gyrus and left posterior cingulate cortex were significantly related to PES. The medial orbitofrontal cortex is an important part of the human pleasure system. A recent meta-analysis of 87 neuroimaging studies found that the medial orbitofrontal cortex is associated with characterization and pleasure in a series of sensations (such as taste, hearing, and tactile feeling) (46–48). The connection between the dorsomedial prefrontal cortex and medial orbitofrontal cortex was positively related to PES. This may indicate that individuals who are adept at sharing others' positive emotions may have a stronger relationship between the self-information representation system and the reward system in the brain. This may make it easier for them to convert others' happiness into their own pleasure. The posterior cingulate cortex connects many cortical and subcortical areas and is therefore considered an association cortex. This area allows the brain to integrate internal and external information (49). In general, the posterior cingulate cortex is a region for integration of information from the whole brain, and includes not only self-related, but also social-related, information.

We also found that the ALFFs in the right subgenual cingulate were negatively correlated with TPE. Previous brain imaging studies have shown that the subgenual cingulate (SGC) has important roles in the experience of negative mood states (50, 51), such as guilt and self-directed negative emotional responses to the belief that one has violated a moral norm (52, 53). In clinical studies, the SGC is often considered as a critical hub within distributed networks mediating depression (54, 55). For example, the volume of SGC is reduced in certain groups of patients with depression (56, 57). Furthermore, decreases in the activity of SGC have been reported following successful treatment of depression with a variety of non-surgical interventions (58, 59). The negative relationship of the ALFFs between the subgenual cingulate and PES maybe indicated that individuals with higher PES have better emotion regulation abilities. This may then allow these subjects to regulate negative emotional experiences and maintain and update positive information. This further validates our previous findings and reflects an important processing characteristic of trait positive empathy.

Functional connection analysis also found that the right subgenual cingulate and the left SMA were significantly related to PES. The supplementary motor area and the prefrontal cortex and cingulate gyrus have a wide range of fiber connections. These areas are not only involved in the implementation of motor function and regulation, but are also important areas for the implementation of control, conflict resolution, and other advanced cognitive functions (60). In a previous diffusion tensor imaging study, it was found that the anisotropy fraction of white matter in the SMA was increased in the early stages of depression intervention, and that improved white matter integrity in the SMA is correlated with depressive symptom remission (61). Qu et al. (62) also report that the spontaneous functional connectivity between the subgenual cingulate cortex and left supplementary motor area is significantly enhanced in patients with major depressive disorder after 4 weeks of psychotherapy. Based on a previous study, we speculated that individuals with positive empathy traits can adjust the cognitive and psychomotor functions of the brain by enhancing the functional connection between the subgenual anterior cingulate cortex and the SMA. This may then lead to better ability to process positive emotions and suppress negative emotions.

Taken together, our results provide neural evidence for our hypothesis that TPE and TNE might be two very similar but independent psychological structures. First, consistent with trait negative empathy in traditional research, the level of positive empathy is also closely related to the emotional ability of the individual. Those who are adept at sharing the positive emotions of others are more adept at inferring the mental state of others and resonating with them. Second, trait positive empathy also has its own characteristic cognitive neural mechanisms. Individuals with higher level of trait positive empathy may have less self-centered tendencies, be better at processing positive emotional information, and have higher levels of negative emotional regulation. The results of this study may be significant for the cultivation of trait positive empathy. Sharing the emotions of others has been promoted as a good personality quality in different cultural contexts. According to the results of this study, learning to think of others in interpersonal communication, management of negative emotions, and more optimistic attitudes may be important for the cultivation of this quality.

Although the current study provided evidence novel information the neural correlation with TPE, there are still several limitations need to be considered. First, the main concern about this study is the validity of measuring one's TPE. It should be more intriguing if the author compared the neural relevance differences between TPE and TNE, which could further explored in future research. Next, the present study is only a related study, which cannot clearly show the causal relationship between neural correlates of trait positive empathy, more sophisticated experimental design in the future can be used to study the causal relationship. Finally, our subjects are college students or graduate students with higher education level, and their age range is relatively small, so there are some limitations on the generalizability of our results interpretation.

## Conclusion

To sum up, we investigated whether individual differences in TPE are reflected in spontaneous brain activity in the resting state, as assessed using ALFFs and RSFC. We found that higher PES was associated with higher ALFFs in the right insula and lower ALFFs in the right subgenual cingulate, right dorsomedial prefrontal cortex, and right precuneus. These brain regions form functional networks with other brain regions that together predict TPE levels. The above brain resting state spontaneous activity level data indicate that individuals who are adept at sharing the positive emotions of others are more sensitive not only to emotional stimuli from others, but also to the processing of positive emotional information. These individuals are also able to effectively suppress the influence of negative emotional information. Furthermore, individuals with higher trait positive empathy may also have less self-centered tendencies. These findings have important implications for our understanding of the nature of TPE and the implementation of appropriate interventions.

## Data Availability Statement

Publicly available datasets were analyzed in this study. This data can be found here: https://doi.org/10.6084/m9.figshare.12928859.

## Ethics Statement

The studies involving human participants were reviewed and approved by Ethics Committee of Southwest University. The patients/participants provided their written informed consent to participate in this study.

## Author Contributions

TY and AF designed experiments. TY and JZ carried out experiments, analyzed sequencing data, and wrote the manuscript. All authors contributed to the article and approved the submitted version.

## Conflict of Interest

The authors declare that the research was conducted in the absence of any commercial or financial relationships that could be construed as a potential conflict of interest.
